# Câncer e Doenças Cardiovasculares na Pandemia de COVID-19

**DOI:** 10.36660/abc.20200405

**Published:** 2020-09-18

**Authors:** Lucas Tokio Kawahara, Isabela Bispo Santos da Silva Costa, Cecília Chie Sakaguchi Barros, Gabriel Coelho de Almeida, Cristina Salvadori Bittar, Stephanie Itala Rizk, Laura Testa, Camila Motta Venchiarutti Moniz, Juliana Pereira, Gláucia Maria Moraes de Oliveira, Maria Del Pilar Estevez Diz, Patricia Oliveira Guimarães, Ibraim Masciarelli Pinto, Roberto Kalil, Ludhmila Abrahão Hajjar, Paulo M. Hoff

**Affiliations:** 1 Faculdade de Medicina Universidade de São Paulo São Paulo SP Brasil Faculdade de Medicina da Universidade de São Paulo (FMUSP), São Paulo , SP – Brasil; 2 Universidade de São Paulo Instituto do Câncer do Estado de São Paulo São Paulo SP Brasil Universidade de São Paulo Instituto do Câncer do Estado de São Paulo , São Paulo , SP – Brasil; 3 Hospital Sírio-libanês São Paulo SP Brasil Hospital Sírio-libanês , São Paulo , SP - Brasil; 4 Universidade de São Paulo Instituto do Coração São Paulo SP Brasil Universidade de São Paulo Instituto do Coração – Cardio-Oncologia, São Paulo , SP - Brasil; 5 Instituto D’Or de Pesquisa e Ensino São Paulo SP Brasil Instituto D’Or de Pesquisa e Ensino , São Paulo , SP - Brasil; 6 Universidade Federal do Rio de Janeiro Rio de Janeiro RJ Brasil Universidade Federal do Rio de Janeiro , Rio de Janeiro , RJ - Brasil; 7 Instituto Dante Pazzanese de Cardiologia São Paulo SP Brasil Instituto Dante Pazzanese de Cardiologia , São Paulo , SP - Brasil; 8 Grupro Fleury Medicina Diagnóstica São Paulo SP Brasil Grupro Fleury Medicina Diagnóstica , São Paulo , SP - Brasil

**Keywords:** Coronavírus, COVID-19, Betacoronavirus/complicações, Doenças Cardiovasculares/complicações, Síndrome Respiratória Aguda Grave, SARS-CoV-2, Pandemia, Neoplasias/complicações, SARS-CoV-19

## Abstract

O desafio imposto ao sistema de saúde pela pandemia da COVID-19 faz com que haja uma necessidade de readequações de rotinas e serviços de saúde, com os objetivos de controlar a disseminação do vírus e preservar a saúde. Torna-se ainda mais importante o manejo seguro e correto dos pacientes dos grupos de risco, como os pacientes idosos, os portadores de doenças cardiovasculares e os pacientes com câncer. Dessa forma, a cardio-oncologia ganha novo dimensionamento, no intuito de se adequar às necessidades dos pacientes diante de uma pandemia, reestruturando o sistema de atendimento de forma a oferecer qualidade e segurança na assistência à saúde.

## Introdução

A *coronavirus disease 2019 (COVID-19),* cujo agente patogênico foi descrito como um betacoronavírus de RNA envelopado, nomeado de *severe acute respiratory syndrome coronavirus 2 (SARS-CoV-2)* , teve seus primeiros casos descritos em dezembro de 2019 e rapidamente se disseminou pelo mundo, tendo sido denominada pela Organização Mundial da Saúde (OMS) como uma pandemia em 11 de março de 2020. ^1^

Os dados epidemiológicos atuais mostram que pacientes com câncer e doenças cardiovasculares (DCV) são frequentemente acometidos e apresentam pior evolução quando infectados pela COVID-19. A incidência e a mortalidade por complicações cardiovasculares são maiores nesses pacientes. ^2 - 4^ O número crescente de pacientes e a sobrecarga nos serviços de saúde em áreas nas quais o risco de contágio é elevado geraram a necessidade de se discutir adequações nas rotinas de cuidados desses pacientes visando preservá-los, sem causar impacto no tratamento de suas comorbidades. ^5 - 7^

Dessa forma, esta revisão tem por objetivo sistematizar o atendimento dos serviços de cardio-oncologia quanto ao manejo do paciente com câncer e DCV durante a pandemia, buscando adotar o tratamento necessário com a máxima segurança.

### Covid-19 em Pacientes com Câncer

Pacientes com câncer ativo ou em remissão, demonstraram-se como um notável grupo de risco à infecção pelo SARS-CoV-2. Vários aspectos corroboram para o enquadramento dos pacientes oncológicos como um grupo de maior vulnerabilidade relacionada à infecção pelo SARS-CoV-2, com maior chance de evolução para formas graves, com velocidade mais rápida de deterioração e óbito. ^3^ O risco aumentado em pacientes oncológicos pode estar relacionado ao estado de imunossupressão sistêmica que pode ser atribuído tanto aos tratamentos antineoplásicos (quimioterapias e cirurgias para ressecção tumoral) como também à própria malignidade tumoral e ao mesmo tempo ao aumento de DCV nessa população. ^8 , 9^

A prevalência de câncer nos estudos epidemiológicos que descrevem pacientes com COVID-19 é bastante variável. ^10 , 11^ Estudos chineses mostram prevalência de 1%, número consideravelmente mais elevado que a prevalência de câncer nesse país (0,29%). ^2^ Em análise de 5.700 casos em Nova York, 6% dos pacientes tinham câncer. ^11^ Grasselli et al. em coorte italiana com 1.571 pacientes demonstraram que câncer era uma das comorbidades mais prevalentes, correspondendo a 8% de todos os pacientes analisados. ^12^

Em estudo recentemente publicado, quando comparados a paciente sem câncer, os pacientes com câncer e COVID-19 eram mais idosos (63,1 anos [± 12,1%] vs. 48,7 anos [± 16,2]), mais frequentemente tabagistas (2 [22%] vs. 107 [7%]) e tinham alterações mais graves na tomografia computadorizada (TC) de tórax (17 [94%] vs. 1113 [71%]). ^3^ Não foram observadas diferenças quanto a sexo ou outras comorbidades. ^3^ Os pacientes com câncer apresentaram maior taxa de complicações (7 [39%] vs. 124 [8%]). ^3^

A idade avançada é preditor independente de gravidade em pacientes com COVID-19. A mediana de idade dos pacientes que evoluíram a óbito na Itália foi de 81 anos (IQR 73 – 86). ^13^ Zhou et al., ^4^ observaram que na análise multivariada a chance de óbito foi maior (OR 1,10; 95% CI 1,03 – 1,17) para cada ano a mais. ^4^ Nos pacientes com câncer, Liang et al. observaram que a idade avançada foi o único preditor independente da forma grave da doença (OR 1,43; 95% CI 0,97 – 2,12). ^3^ Estes dados sugerem que seja dada especial atenção aos pacientes com idade avançada e câncer, pela maior gravidade observada nesta população.

No grupo com histórico de câncer, foi observada uma heterogeneidade associada ao intervalo de tempo desde a última intervenção terapêutica (cirúrgica ou quimioterápica). Os pacientes que haviam passado por esses procedimentos no mês anterior à infecção pelo SARS-CoV-2 apresentaram maior incidência de eventos graves do que os sobreviventes de câncer que não tinham sido submetidos a nenhuma intervenção terapêutica, sugerindo a associação entre o estado de imunossupressão causado pelo tratamento antitumoral e o maior risco de gravidade na apresentação da COVID-19. ^3^

Os dados iniciais de mortalidade de pacientes com câncer e COVID-19 sugerem que estes casos têm mortalidade elevada em comparação aos pacientes sem câncer. Estudos de coortes italianas mostram uma prevalência de 16% a 20% de pacientes com câncer ativo dentre os pacientes que vieram a óbito pela COVID-19. ^13 , 14^ Pacientes com câncer apresentam taxa de letalidade de 7,6% em virtude da COVID-19, substancialmente maior do que a taxa de letalidade de 1,4% de pacientes sem condições de comorbidades. ^9^

Dessa maneira, conhecendo os riscos que esses pacientes correm ao serem infectados pelo SARS-CoV-2, é essencial que pacientes com câncer sejam constantemente acompanhados e monitorados em locais em que casos de COVID-19 estejam em ascensão. Este acompanhamento pode utilizar o auxílio da telemedicina. ^6^

### Definição do Tratamento Oncológico

Muito se discute a respeito do adiamento das intervenções diagnósticas e terapêuticas nos pacientes com câncer durante o período da pandemia. As visitas frequentes aos hospitais poderiam aumentar o risco de contágio desses pacientes e da equipe de saúde. ^6^ As medidas de distanciamento social têm sido incentivadas no intuito de diminuir a disseminação da COVID-19, uma vez que a transmissão é elevada e ocorre mesmo por pessoas assintomáticas. ^15^ Em contrapartida, sabe-se que muitos tipos de câncer apresentam morbidade e mortalidade mais elevadas que a COVID-19 e o adiamento de terapia tem implicações prognósticas importantes, ^16^ especialmente em locais onde os recursos de saúde são habitualmente limitados e a espera pelo atendimento oncológico pode demorar meses. Dessa forma, uma avaliação criteriosa que leve em consideração o tipo de câncer, o *status* da performance do paciente e o tipo de terapia antineoplásica necessária são extremamente relevantes para se fazer um balanço entre riscos e benefícios do possível adiamento do tratamento antitumoral. ^17^

Recomenda-se iniciar ou continuar terapias adjuvantes e neoadjuvantes (ou qualquer outra terapia com potencial curativo), bem como manter terapia para doenças metastáticas nas quais o benefício do tratamento seja claro na literatura. ^6^ Procedimentos cirúrgicos oncológicos com potencial curativo continuam a ser recomendados, avaliando sempre a morbidade cirúrgica do procedimento e a existência de outras terapias neoadjuvantes mais seguras. ^18^

Algumas estratégias têm sido discutidas como alternativas benéficas durante a pandemia, especialmente para pacientes idosos em locais com transmissão elevada. A escolha da terapia oncológica deve levar em consideração as opções locais disponíveis, evitando que pacientes façam longos deslocamentos (viagens intermunicipais) até o serviço de saúde. Deve-se dar preferência à realização de quimioterapia oral quando indicada e à endocrinoterapia em tumores sensíveis. ^5^

Pode-se ainda considerar, a depender da resposta do paciente, tipo do tumor e tempo da terapia, espaçar ciclos de pacientes em imunoterapia de duas ou três para quatro ou seis semanas. ^5^ Em pacientes em radioterapia, para tratamento de doenças mais comuns (como mama, próstata, pulmão e cabeça), discute-se a redução do fracionamento da dose, na qual utiliza-se dose mais elevada e menor número de sessões. Esta abordagem pode levar ao aumento da toxicidade, e dessa forma, devem ser sempre discutidos o risco e o benefício do hipofracionamento. ^19^

É importante que toda estratégia seja discutida e debatida com o paciente e familiares. A relação médico paciente é extremamente importante nesse momento e o auxílio da telemedicina pode aproximar esta interação e diminuir a exposição do paciente. ^6 , 17^ Recomenda-se ainda que, se possível, existam serviços de saúde direcionados para o tratamento oncológico, que não sejam referências para receber pacientes com a COVID-19. As recomendações realizadas pela *American Society of Clinical Oncology* referentes a cada tipo de procedimento específico em pacientes oncológicos estão resumidas na Tabela 1 . ^20^

**Tabela 1:** – Orientações quanto aos procedimentos em pacientes oncológicos durante a pandemia da COVID-19

Período	Procedimentos	Recomendações
**Pré-diagnóstico**	Rastreamento oncológico	- Procedimentos de rotina em pacientes assintomáticos que requisitem visitas a centros de saúde, como mamografias e colonoscopias, devem ser postergados. - Pacientes com suspeita de doença devem ser investigados. - Biópsias e exames de imagem em pacientes com suspeita de neoplasia devem ser realizados.
**Diagnóstico oncológico ativo**	Estadiamento	- Avaliação criteriosa de adiamento considerando doenças de comportamento indolente.
	Radioterapia	- Em pacientes com tumores de progressão rápida ou em pacientes nos quais a radioterapia curativa tem benefício claro, a radioterapia deve ser mantida; - Alguns pacientes podem ter sua radioterapia adiada de forma segura, porém discussões multidisciplinares são fundamentais na tomada de decisão individual.
	Imunoterapia	- A decisão clínica de postergar as terapias imunossupressoras deve ser tomada de maneira individualizada, considerando os seguintes fatores: • risco de recorrência do câncer caso haja alguma mudança na rotina de terapia; • número de ciclos da terapia completados; • tolerância do paciente ao tratamento. - Em pacientes com tumores não estáveis, não se recomenda suspender a quimioterapia.
	Quimioterapia	
	Cirurgia oncológica	- Deve ser feita rotineiramente uma análise de riscos do dano em virtude do adiamento da cirurgia e dos benefícios da cirurgia, em meio a pandemia pela COVID-19.
	Transplante de célula-tronco	- Pacientes em alto risco para a COVID-19 cuja neoplasia possa ser temporariamente controlada com terapia convencional podem ter o transplante de célula-tronco adiado. - Apesar da falta de evidências quanto à transmissão do SARS-CoV-2 via transfusão sanguínea, é prudente testar os potenciais doadores para COVID-19.
**Diagnóstico oncológico em remissão**	Rotina de seguimento	- Em casos de pacientes com baixo risco de recorrência e assintomáticos no período de acompanhamento, pode-se postergar a rotina de seguimento para evitar as visitas clínicas e a possível exposição; - A telemedicina é ferramenta útil para que a avaliação dos pacientes seja feita de maneira segura e regular; - Nos casos em que haja uma recomendação de uma frequência de intervenção em um paciente, adiar a rotina de intervenção para a menor frequência recomendada é prudente.

### Avaliação Prognóstica

O manejo do paciente com câncer deve incluir a discussão de cuidados paliativos naqueles cuja expectativa de vida seja limitada. No contexto dessa pandemia, essa discussão ganha ainda mais importância, evitando-se a exposição desses pacientes com prognóstico ruim ao contágio da COVID-19. É importante a discussão de diretivas antecipadas com os pacientes portadores de doenças crônicas terminais, sempre respeitando as regulações locais, para que estes não sejam submetidos a terapia de cuidados intensivos (ventilação mecânica, procedimentos invasivos, reanimação cardiopulmonar), caso essa opção seja considerada a mais adequada e proporcional dentro do contexto da doença de base. ^21^

### Interseção entre Câncer, Doença Cardiovascular e a COVID-19

As DCV e o câncer são as duas principais causas de morte nos países desenvolvidos e em desenvolvimento, apesar de melhorias significativas na prevenção, triagem e tratamento de ambas as doenças. ^16 , 22^ Com muitos fatores de risco em comum, o câncer e as DCV frequentemente coexistem nos mesmos indivíduos; aqueles diagnosticados com câncer de pulmão, câncer de mama e câncer de cólon têm maior risco de DCV e aqueles com DCV têm maior risco de desenvolver muitos tipos de câncer. ^22^ A prevalência de fatores de risco cardiovasculares nos pacientes com câncer é elevada, sendo que 60,4% dos pacientes têm hipertensão, 23,9% têm diabetes e 22,4% tem dislipidemia. ^23^

O manejo dos pacientes com câncer e DCV sob tratamento oncológico é multidisciplinar, tendo como metas o controle de fatores de risco, a redução de complicações cardiovasculares e a minimização de interrupções desnecessárias do tratamento oncológico. As terapias antineoplásicas são potencialmente tóxicas ao coração e a incidência de complicações cardiovasculares em sobreviventes é elevada. ^24^ A interação entre as especialidades oncológicas (clínica e cirúrgicas) e o serviço de cardio-oncologia é fundamental para o manejo adequado desses pacientes. ^23 , 25^

**Figura 1:**
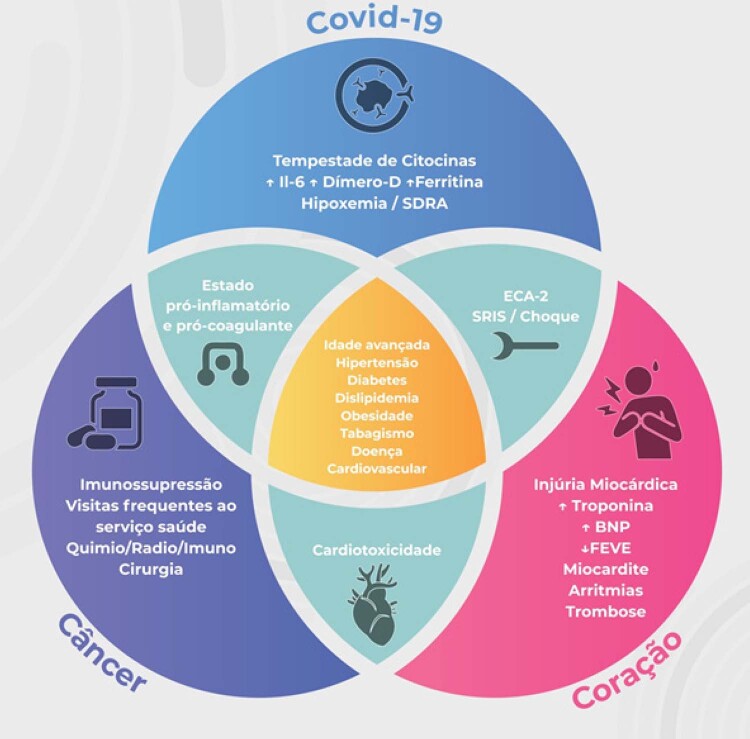
– Interseção entre o câncer, o coração e a COVID-19. A hipertensão, idade avançada, diabetes, obesidade e tabagismo são fatores de risco comuns às três doenças. O manejo no câncer na COVID-19 visa evitar visitas desnecessárias aos serviços de saúde, uma vez que estes pacientes sob tratamento oncológico são imunossuprimidos e mais suscetíveis ao desenvolvimento de complicações da COVID-19. As formas graves da COVID-19, manifestadas por hipoxemia e síndrome do desconforto respiratório agudo, decorrem de processo inflamatório sistêmico, com elevações de citocinas inflamatórias e fatores pró-trombóticos. As complicações cardiovasculares são frequentes nessa população, como trombose, arritmias, miocardite e disfunção ventricular. ECA2: enzima conversora de angiotensina 2; FEVE: fração de ejeção do ventrículo esquerdo; IL-6: interleucina-6; SDRA: síndrome do desconforto respiratório agudo; SRIS: síndrome da resposta inflamatória sistêmica. Quimio: quimioterapia; Radio: Radioterapia; Imuno: imunoterapia.

Esse cuidado integral ganha ainda mais sentido em pacientes com a infecção pelo SARS-CoV-2, que agrega morbimortalidade considerável. A interseção entre as três doenças vai desde o controle de fatores de riscos, quanto ao manejo das complicações pulmonares e cardiovasculares, bem como o ajuste do tratamento oncológico e prevenção de eventos tromboembólicos. A Figura 1 ilustra os principais pontos de ligação entre essas três doenças. O conhecimento e a implementação precoce de medidas terapêuticas adequadas podem resultar em melhora do prognóstico dos pacientes com câncer.

### A COVID-19 e as Doenças Cardiovasculares

A presença de DCV preexistente é apontada como um dos principais fatores de risco para ocorrência e gravidade da COVID-19 (9). Uma meta-análise recentemente publicada, abrangendo um total de 1.527 pacientes, observou que em pacientes com COVID-19, as prevalências de hipertensão, doenças cardíacas e cerebrovasculares, e diabetes eram de 17,1%, 16,4% e 9,7%, respectivamente. ^26^ Richardson et al. em uma coorte estadunidense com 5.700 pacientes observaram que as comorbidades mais prevalentes foram hipertensão (3026 [56,6%]), obesidade (1737 [41,7%]) e diabetes (1808 [33,85%]). ^11^ Em uma coorte italiana com 1.591 pacientes, hipertensão (509 [49%]) foi a comorbidade mais prevalente, seguida de DCV (223 [21%]), dislipidemia (188 [18%]) e diabetes (180 [17%]). ^12^

Além da prevalência elevada, estes pacientes têm maior predisposição para o desenvolvimento de formas graves da doença e sua evolução a óbito. Em estudo chinês, a mortalidade de pacientes com DCV foi de 10,5%, sendo nos diabéticos 7,3% e nos hipertensos 6,0%, taxas estas maiores que nos pacientes sem comorbidades (2,3%). ^27^ Dos pacientes que vieram a óbito na Itália, 3,6% não tinham comorbidades, 14,4% uma única comorbidade, 21,1% duas, e 60,9% três ou mais, sendo hipertensão (69,1%), diabetes (31,7%) e DCV (27,5%) as comorbidades mais prevalentes. ^13^ Em estudo publicado por Jianfeng Xie et al., com 168 pacientes que morreram pelo novo coronavírus **,** 74,4% tinha alguma comorbidade. ^28^ As comorbidades mais comuns foram hipertensão (50%), diabetes (25%) e cardiopatia isquêmica (18,5%). ^28^

O SARS-CoV-2 causa lesão ao sistema cardiovascular por diferentes mecanismos. O vírus utiliza a proteína de membrana enzima conversora de angiotensina 2 (ECA 2) no início da ligação do vírus com o hospedeiro. ^29^ A ECA2 modula negativamente o sistema renina-angiotensina-aldosterona por meio da conversão de angiotensina 2 em angiotensina 1-7, o que se opõe à ação da enzima conversora de angiotensina (ECA). ^30^ Ela é altamente expressa em tecidos pulmonares e cardíacos, e exerce funções importantes de proteção cardiovascular e pulmonar. ^9 , 31^ A ligação viral a essa proteína de membrana causa inibição desses mecanismos de proteção, podendo resultar em inflamação do miocárdio, edema pulmonar e insuficiência respiratória aguda. ^32 , 33^

A lesão cardiovascular pode ainda decorrer da resposta inflamatória sistêmica, que resulta no fenômeno de tempestade de citocinas. Nesse sentido, em casos mais graves, a infecção resultaria em uma resposta desbalanceada por células Th1 e Th2. ^34^ A elevação de interleucina-6 (IL-6) foi apontada como um dos preditores de mortalidade na COVID-19, sugerindo que a hiperinflamação em resposta à infecção pelo SARS-CoV-2 seja um fator importante de mortalidade. ^35^

As principais complicações cardiovasculares resultantes da COVID-19 são injúria miocárdica, insuficiência cardíaca, miocardite, arritmias cardíacas, choque e insuficiência coronária. ^36^ Em alguns estudos, a elevação isolada de troponina foi preditor independente de mortalidade ( *OR* = 26.909, 95% *CI* 4.086 – 177.226).37 Guo et al., observaram que entre 187 pacientes com COVID-19, 52 (27,8%) tinham lesão miocárdica e a mortalidade foi marcadamente maior em pacientes com níveis elevados de troponina T do que em pacientes com níveis normais de troponina T (59,6% vs. 8,9%).38 Níveis aumentados de dímero-D também tem relação direta com mortalidade.4 A injúria miocárdica esteve relacionada a quadros graves da doença, com maior necessidade de internação em unidade de terapia intensiva (UTI), disfunção ventricular e choque.4,39

Os casos de disfunção ventricular observados nos pacientes com COVID-19 são resultados de síndrome coronariana aguda (SCA), miocardite e síndrome de takotsubo. ^40 , 41^ A SCA com elevação do segmento ST nos pacientes com COVID-19 acontece mesmo sem doença arterial coronariana (DAC) obstrutiva, sendo necessária avaliação criteriosa desses pacientes com avaliação da função ventricular e dos níveis de biomarcadores. Em uma coorte com 18 pacientes, 10 (55%) apresentaram supra desnivelamento do segmento ST sem doença coronária obstrutiva, e nestes pacientes a mortalidade chegou a 90%, sendo decorrente possivelmente de inflamação, vasoespasmo, hipóxia e trombose de microcirculação. ^42^

As arritmias cardíacas na COVID-19 podem ser consequência dos quadros de miocardite aguda, injúria miocárdica e de efeitos colaterais da terapia farmacológica. ^38 , 39 , 43^ Em um estudo conduzido com 138 pacientes hospitalizados, 16,7% apresentaram arritmias, sendo mais prevalentes em pacientes internados em UTI (44,4% vs. 6,9%). ^39^ Guo et al., ^38^ observaram que 11,7% apresentavam arritmias malignas (taquicardias ventriculares ou fibrilação ventricular). ^38^ Essas arritmias eram significativamente mais comuns em pacientes com níveis séricos elevados de troponina cardíaca T e de fragmento N-terminal do peptídeo natriurético tipo B. ^38^

Como a COVID-19 é uma doença recente, não se sabe ao certo quais seriam suas consequências cardíacas a longo prazo. Entretanto, constata-se, em um estudo de 12 anos de seguimento, que sobreviventes da infecção por SARS-CoV apresentaram índices aumentados de anormalidades cardiovasculares e de distúrbios no metabolismo de lipídios e glicose. ^44^

Embora os mecanismos de tais alterações não tenham sido explicitados, devido à semelhança estrutural entre o SARS-CoV e o SARS-CoV-2, aventa-se a possibilidade de que a COVID-19 possa ter implicações semelhantes a longo prazo. ^45^

Manejo Clínico de Pacientes com Câncer e Doenças Cardiovasculares em Áreas de Alta Transmissão de COVID-19

**Prevenção.** As medidas de prevenção são etapa primordial no cuidado dos pacientes com câncer e DCV durante a pandemia da COVID-19. A associação de DCV e câncer aumenta o risco da COVID-19 e a gravidade da doença. Considerando todos os aspectos relacionados à quarentena, ao isolamento social e ao distanciamento, a prevenção cardiovascular deve ser reforçada nesses pacientes, com a adoção de medidas de controle da pressão arterial, dos níveis de glicemia e de lipídios, adoção de dieta adequada e o estímulo à realização de atividade física.

Como ainda não há vacina comprovadamente eficaz para o SARS-CoV-2, recomenda-se a atualização do cartão vacina contra influenza e antipneumocócica como forma de diminuir a incidência de infecções sobrepostas. ^5 , 46^

Medidas necessárias para frear a disseminação do vírus devem ser ainda mais enfáticas nessa população, como a higienização correta das mãos com sabão ou álcool em gel, assim como recomendações de evitar o contato com pessoas sintomáticas, bem como aglomerações. O Ministério da Saúde, em concordância com a OMS, aconselha que todos devam higienizar frequentemente as mãos e os objetos de uso frequente, além de minimizar o compartilhamento de objetos e evitar levar as mãos aos olhos, boca e nariz. ^47^

Em locais de risco elevado, medidas de minimização de contato e de exposição devem ser tomadas nos casos de maior vulnerabilidade. Nesse sentido, o acompanhamento e a visita dos pacientes com DCV e/ou câncer estável pode ser feito com o auxílio da telemedicina, evitando a transmissão do SARS-CoV-2 a esse grupo de risco. ^48^ A telemedicina foi recentemente regularizada pelo Conselho Federal de Medicina durante a pandemia e deve ser utilizada nesses pacientes a fim de reduzir as idas frequentes aos serviços. As consultas podem ser feitas via telefone ou vídeo-consulta, visando especialmente controle de fatores de risco cardiovasculares (como hipertensão, diabetes, tabagismo, obesidade, dentre outros) e monitorização de sintomas.

Para pacientes com histórico recente de transplante de células-tronco, o auto-isolamento pode ser uma maneira eficaz de evitar exposição em áreas de risco elevado. Pessoas em contato com esses pacientes também precisam aderir às medidas de precauções necessárias, minimizando a exposição ou obtendo testes para SARS-CoV-2 quando apresentarem sintomas gripais.

Pelas alterações imunológicas, pacientes em tratamento para neoplasias podem apresentar um quadro atípico de COVID-19, com sintomas mais brandos mascarando quadros graves. Pacientes em tratamento oncológico e seus médicos devem ter atenção redobrada para quaisquer sintomas.

As medidas de prevenção envolvem também a organização dos serviços de saúde para o atendimento correto desses pacientes. Idealmente, os hospitais dos pacientes com câncer não devem ser os mesmos que atendem casos com suspeita/confirmação de COVID-19. Caso exista essa possibilidade, recomenda-se que haja um serviço direcionado para atendimento destes pacientes. ^47 , 49^ Em caso de suspeita de COVID-19, estes pacientes devem ter seu atendimento priorizado pois apresentam rápida progressão para formas graves. ^3^ Caso não haja um serviço referenciado a eles, recomenda-se organizar fluxo seguro de atendimento a esses pacientes nas clínicas e hospitais, com a adoção de todas as medidas de prevenção.

**Figura 2:**
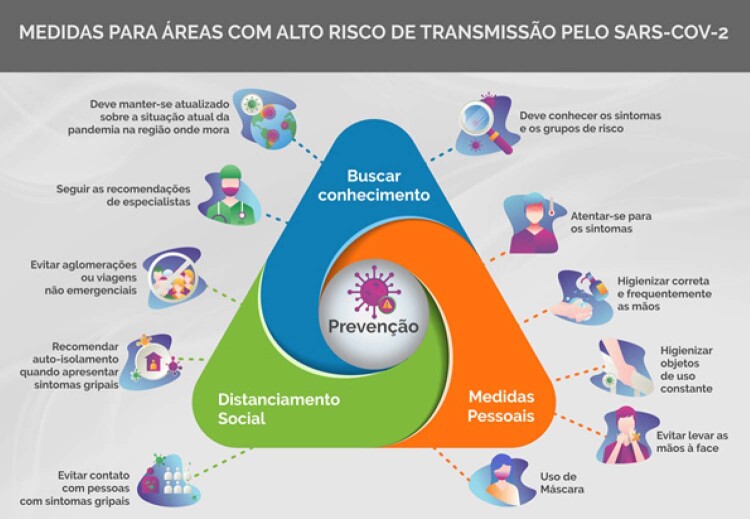
– Medidas de prevenção para a população geral em área com alto risco de infecção por SARS-CoV-2. SARS-CoV-2: severe acute respiratory syndrome coronavirus 2.

É importante também que os profissionais de saúde envolvidos no atendimento desses pacientes tomem os cuidados adequados com relação a medidas de prevenção. É recomendado o uso de equipamentos de proteção individual, higienização das mãos antes e depois do contato com o paciente ou materiais de uso do paciente, bem como o cuidado na manipulação de fármacos a serem empregados ( Figura 2 ). ^6^

Pacientes com câncer já são submetidos a grande estresse psicológico em resultado do próprio diagnóstico. Dessa forma, aconselha-se que estes pacientes recebam informações claras, apoio de familiares e sejam estimulados a terem um estilo de vida saudável durante a quarentena, com prática de exercício físico e alimentação saudável. Isso pode auxiliar a reduzir ansiedade e estresse emocional. As medidas de distanciamento social são importantes para prevenção da disseminação da COVID-19, entretanto podem causar prejuízo à saúde mental dos pacientes com quadro de ansiedade. ^50^ Um recente trabalho sobre o impacto psicológico da quarentena indica a existência de tensão psicológica sobre aqueles que não podem ou não são capazes de participar da vida social. ^51^ É importante ressaltar a todos os pacientes com câncer que havendo urgência ou complicações relacionadas ao câncer ou ao sistema cardiovascular, os mesmos devem procurar os serviços de emergência imediatamente, evitando-se maus resultados relacionados ao receio de procurar assistência de saúde.

### Conduta em Relação a Exames Diagnósticos e a Terapia nos Pacientes com Câncer

*Exames diagnósticos* **:** Os pacientes com câncer frequentemente necessitam da realização de exames para diagnóstico, estadiamento e avaliação de resposta terapêutica. O monitoramento varia a depender do diagnóstico oncológico, mas, muitas vezes, é necessária a realização de marcadores tumorais séricos, TC, cintilografia óssea e tomografia por emissão de pósitrons (PET *,* siglas em inglês de *positron emission tomography)* .

A realização dos exames de medicina nuclear (cintilografia e PET) demandam tempo prolongado e vários profissionais envolvidos. A exposição do paciente e dos profissionais de saúde ao contágio da COVID-19 é, por isso, relativamente elevada, sugerindo-se que a indicação da realização desses exames, durante o período da pandemia, seja discutida e individualizada, avaliando-se os riscos e os benefícios para cada paciente. Recomenda-se o adiamento dos exames para, pelo menos, 14 dias em pacientes suspeitos ou confirmados como portadores da infecção pelo novo coronavírus. ^52^

A realização de TC deve ser priorizada no contexto atual para avaliação da infecção por COVID-19, e da presença de tromboembolismo pulmonar. ^49^ Os achados tomográficos da COVID-19 são variáveis, sendo mais comuns as imagens reticulares e infiltrado em vidro fosco periféricas e bilaterais. ^49^ Recomenda-se evitar a realização de ecocardiograma transesofágico e de cintilografia pulmonar durante a pandemia pelo elevado risco de contaminação. ^7 , 52^ A realização de ressonância magnética cardíaca deve ser feita após cuidadosa avaliação do paciente e da indicação clínica, uma vez que pela maior duração e pelas características deste tipo de exame, tanto o risco de contágio como a limpeza do equipamento têm maior complexidade. ^53^ O Centro de Controle de Doenças estadunidense sugere cautela na indicação deste tipo de investigação e sociedades internacionais recomendam que sua realização obedeça a protocolos rigorosos. ^53^

Durante o período da pandemia, recomenda-se que aqueles pacientes que estejam em remissão, sem doença oncológica ativa e estejam assintomáticos possam ter seus exames de controle reagendados para momento oportuno, após a pandemia, ^6^ Adicionalmente, deve-se priorizar a realização de exames em pacientes com indicações urgentes e que necessitem iniciar tratamento com potencial curativo. ^6^ Pacientes com idade superior a 65 anos, ou na presença de comorbidades e com fatores para imunossupressão (neutropenia, neoplasias hematológicas, quimioterapia nos últimos 30 dias, transplantados e em uso de medicações imunossupressoras) devem ter seus atendimentos priorizados, evitando longas esperas, o que poderia sobrecarregar ainda mais os serviços de saúde. ^53 , 54^

*Rastreio de cardiotoxicidade* **:** Os pacientes sob terapia oncológica têm risco potenciais de desenvolvimento de cardiotoxicidade. O espectro de manifestações da cardiotoxicidade é amplo, sendo os principais tipos: insuficiência cardíaca, arritmias, trombose e DAC. ^55^ Os exames de imagem cardíaca são fundamentais para correto diagnóstico e manejo dos pacientes, especialmente em pacientes com indicação de uso de antraciclina e trastuzumabe. O diagnóstico de cardiotoxicidade por essas drogas é classicamente definido pela queda da fração de ejeção de > 10 pontos percentuais para valores menores de 50%.

Nos últimos anos, diversas estratégias de monitorização e rastreio de toxicidade precoce, com diagnóstico ainda na fase subclínica, foram incorporadas na prática clínica para evitar que esses pacientes desenvolvam disfunção ventricular e insuficiência cardíaca. ^55 - 58^ Lopez-Sendon et al. ^59^ identificaram a incidência de cardiotoxicidade em 37% dos pacientes previamente submetidos a terapia com elevado potencial cardiotóxico e que tiveram monitoramento cardíaco durante a quimioterapia. ^59^ Entretanto, no cenário atual da pandemia, avaliando-se o risco potencial de contaminação por COVID-19 em pacientes com câncer sob quimioterapia, considera-se rediscutir a indicação desses exames, reorganizando-se a marcação de exames de maneira personalizada. ^60^

Os principais fatores de risco de cardiotoxicidade são dose elevada de antraciclina (> 250 mg/m ^2^ ): uso concomitante de terapias cardiotóxicas (radioterapia, ciclofosfamida, trastuzumabe, imunoterapia e antraciclinas): doença cardíaca prévia; presença de dois ou mais fatores de risco cardiovasculares; e extremos de idade (< 18 anos ou > 65 anos). ^55^ A presença de sinais e sintomas de insuficiência cardíaca também é indicativa de risco de cardiotoxicidade. Dessa forma, nesses pacientes com risco elevado de cardiotoxicidade deve-se considerar a realização de investigação cardiovascular.

Quando existir a suspeita de cardiotoxicidade, a avaliação da função ventricular nesses pacientes deve ser feita preferencialmente com a ecocardiografia transtorácica, com exame feito de modo direcionado para elucidar a questão. É importante o uso de equipamentos de proteção individual para diminuir o risco de transmissão do vírus durante o exame. ^61^ Pode-se considerar a realização da pesquisa de biomarcadores cardíacos, caso o paciente tenha visita programada para coleta de exames durante a quimioterapia. ^61^ Entretanto, não se recomenda a solicitação rotineira de biomarcadores para pesquisa de cardiotoxicidade. ^62^

**Figura 3:**
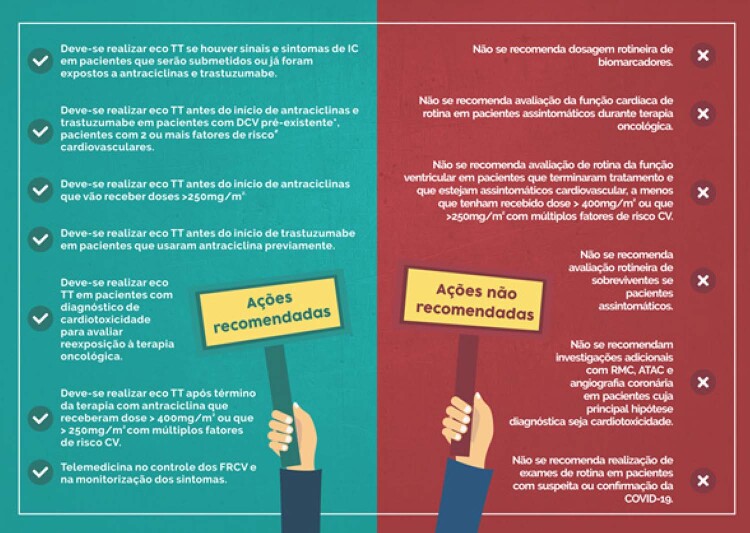
– Recomendações para manejo da cardiotoxicidade durante a pandemia. *Miocardiopatia, doença valvar moderada a importante, arritmia e doença arterial coronariana com revascularização prévia. #Idade > 60 anos, hipertensão arterial, tabagismo, obesidade e dislipidemia. ATAC, angiotomografia de artérias coronárias; DCV: doença cardiovascular; EcoTT, ecocardiograma transtorácico; FRCV: fatores de risco cardiovascular; RMC, ressonância magnética cardíaca.

Outros exames de imagem (angiotomografia coronária, ressonância magnética cardíaca e angiografia coronária) podem ser necessários para investigação adicional de etiologia de disfunção ventricular, uma vez que cardiotoxicidade é diagnóstico de exclusão e as complicações cardiovasculares são frequentes em pacientes com câncer e COVID-19. ^9^ No contexto da exclusão de DAC, a tomografia pode ser particularmente útil na exclusão desta entidade e, assim, dispensar a realização de outros exames, nos quais o risco de contaminação e complexidade são maiores. Recomenda-se a investigação apenas nos pacientes em que a realização do exame deve propiciar modificação da conduta médica. A Figura 3 ilustra as recomendações que visam auxiliar no manejo racional da cardiotoxicidade destes pacientes durante a pandemia.

*Terapia Medicamentosa:* Pacientes com DCV prévias e pacientes que receberam o diagnóstico de cardiotoxicidade (disfunção ventricular) têm indicação de uso de inibidores da enzima de conversão da angiotensina e bloqueadores de angiotensina II. Postulou-se que estes fármacos podem aumentar a expressão dos receptores da ECA2, facilitando a entrada do vírus nas células hospedeiras. Entretanto, evidências mais recentes não confirmaram estes achados e não sugerem que estas medicações aumentem o risco de infecção pela COVID-19. Recomenda-se que essas medicações devam ser mantidas em pacientes com COVID-19, na ausência de contra-indicações. ^63 - 67^ Não se recomenda o uso dessas medicações como profilaxia primária de cardiotoxicidade.

A abordagem do paciente com COVID-19 deve levar em conta a apresentação clínica e a presença de sinais e sintomas de gravidade, como hipoxemia e dispneia. Vários medicamentos foram testados nestes cenários, como imunomoduladores, antivirais, antibióticos, corticosteroides, inibidores de Il-6 e interferon, entretanto, ainda não há dados definitivos sobre a eficácia e a segurança desses fármacos na COVID-19. ^68^

Estudos iniciais sugeriram benefício potencial do uso da cloroquina e hidroxicloroquina na COVID-19, por terem a capacidade de aumentar o pH endossômico das células e reduzir a replicação do SARS-CoV-2. ^69 , 70^ Entretanto, outros estudos não conseguiram corroborar o benefício do uso dessas drogas, e ainda mais, sugerem aumento do risco de mortalidade dos pacientes que receberam cloroquina. ^71 , 72^ Dessa forma, o Ministério da Saúde, pelas evidências apenas incipientes do benéfico dessas drogas, sugere que estes medicamentos possam ser utilizados, em casos confirmados e a critério médico, como terapia adjuvante no tratamento de formas graves, em pacientes hospitalizados, sem que outras medidas de suporte sejam preteridas. ^73^

A linfohistiocitose hemofagocítica (LHH) é uma síndrome hiperinflamatória ainda subdiagnosticada em pacientes com a COVID-19. Esta síndrome é caracterizada como um estado de hipercitocinemia potencialmente fatal que leva a múltipla disfunção orgânica. ^74^ Os principais achados da LHH incluem: febre, citopenias e hiperferritinemia; envolvimento pulmonar (incluindo síndrome do desconforto respiratório agudo) ocorre em aproximadamente 50% dos pacientes. ^74^

Dos pacientes com COVID-19, aqueles que evoluíram a óbito tinham níveis mais elevados de ferritina e Il-6, sugerindo que a inflamação esteja relacionada com a mortalidade desses pacientes. ^75^ Dessa forma, na hiperinflamação observada na COVID-19, a imunossupressão parece ser benéfica. As opções terapêuticas incluem esteróides, imunoglobulina intravenosa, bloqueio seletivo de citocinas (por exemplo, anakinra ou tocilizumabe) e inibição de Janus Kinase. ^74^

### Perspectivas

Espera-se que com a evolução do conhecimento da infecção pelo novo coronavírus, entendamos cada vez mais o comportamento da doença em pacientes com comorbidades como câncer e DCV. As perspectivas em relação a um tratamento eficaz são grandes, e independentemente do tempo necessário para que a ciência abra o caminho para melhores resultados, o oncologista e o cardiologista devem fortalecer ainda mais a linha de cuidado do paciente com câncer. A prevenção cardiovascular, o tratamento adequado do câncer e da DCV, e a análise criteriosa da estratégia diagnóstica e terapêutica são essenciais para a obtenção de bons resultados nessa população frente à pandemia da COVID-19.

## Conclusão

A interseção entre câncer, o coração e COVID-19 é complexa e a racionalização do cuidado desses pacientes é pautada em uma interação entre as especialidades e na personalização de condutas. A avaliação dos riscos e benefícios da realização de intervenções terapêuticas e diagnósticas requer atenção individualizada, considerando-se o prognóstico oncológico e o risco de contágio da COVID-19, especialmente em regiões cuja transmissibilidade esteja elevada. Os pacientes com câncer e com DCV apresentam formas mais graves da infecção pelo novo coronavírus, e por isto, devemos reforçar ainda mais as medidas de prevenção nesses pacientes.
