# Supplementary material for: Câncer e Doenças Cardiovasculares na Pandemia de COVID-19
Source: Arq Bras Cardiol. 2020 Sep 18;115(3):547–57. [Article in Portuguese] doi: 10.36660/abc.20200405 (PMC9363105; doi:10.36660/abc.20200405)
Supplement: Translation

# Translation: Cancer and Cardiovascular Diseases during the COVID-19 Pandemic

**Authors:** Lucas Tokio Kawahara, Isabela Bispo Santos da Silva Costa, Cecília Chie Sakaguchi Barros, Gabriel Coelho de Almeida, Cristina Salvadori Bittar, Stephanie Itala Rizk, Laura Testa, Camila Motta Venchiarutti Moniz, Juliana Pereira, Gláucia Maria Moraes de Oliveira, Maria Del Pilar Estevez Diz, Patricia Oliveira Guimarães, Ibraim Masciarelli Pinto, Roberto Kalil, Ludhmila Abrahão Hajjar, Paulo M. Hoff

**Affiliations:** 1 Brazil Faculdade de Medicina da Universidade de São Paulo (FMUSP), São Paulo, SP – Brazil; 2 Brazil Universidade de São Paulo Instituto do Câncer do Estado de São Paulo, São Paulo, SP – Brazil; 3 Brazil Hospital Sírio-libanês, São Paulo, SP - Brazil; 4 Brazil Universidade de São Paulo Instituto do Coração – Cardio-Oncologia, São Paulo, SP - Brazil; 5 Brazil Instituto D’Or de Pesquisa e Ensino, São Paulo, SP - Brazil; 6 Brazil Universidade Federal do Rio de Janeiro, Rio de Janeiro, RJ - Brazil; 7 Brazil Instituto Dante Pazzanese de Cardiologia, São Paulo, SP - Brazil; 8 Brazil Grupo Fleury Medicina Diagnóstica, São Paulo, SP - Brazil

**Keywords:** Coronavirus, COVID-19, Betacoronavirus/complications, Cardiovascular Diseases/complications, Severe Acute Respiratory Syndrome/complications, Neoplasms/complications, Pandemics, SARS-CoV-19

## Abstract

The challenges that the COVID-19 pandemic cretead to the healthcare system have made it necessary to adapt routines and services, with the objectives of controlling the spread of the virus and preserving health. Safe and correct management of patients in risks groups, such as elderly patients, patients with cardiovascular diseases, and patients with cancer, has become even more important. Thus, cardio-oncology has gained a new dimension, with the aim of adapting to patients’ needs during the pandemic by restructuring the system of care in a manner that offers quality and safety in healthcare.

## Introduction

The first cases of coronavirus disease 2019 (COVID-19), whose pathological agent has been described as an RNA-enveloped betacoronavirus, named severe acute respiratory syndrome coronavirus 2 (SARS-CoV-2), were described in December 2019; the disease rapidly spread throughout the world, and it was declared a pandemic by the World Health Organization (WHO) on March 11, 2020. ^1^

The current epidemiological data show that patients with cancer and cardiovascular diseases (CVD) are frequently affected, and they show worse prognosis when infected with COVID-19. Incidence and mortality due to cardiovascular complications are higher in these patients. ^2 - 4^ The growing number of patients and the overload in health services in areas where the risk of contagion is high have generated the need to discuss adjustments in the care routines for these patients in order to preserve care, without impacting the treatment of comorbidities. ^5 - 7^

Thus, the objective of this review is to systematize the care provided by cardio-oncology services regarding management of patients with cancer and CVD during the pandemic, seeking to adopt the necessary treatment with maximum safety.

### COVID-19 in Patients with Cancer

Patients with cancer, whether active or in remission, have been shown to be a notable risk group for SARS-CoV-2 infection. Several aspects corroborate the classification of patients in oncology as a group of greater vulnerability in relation to SARS-CoV-2 infection, with higher chances of progressing to severe forms and faster rates of deterioration and death. ^3^ The increased risk in patients in oncology may be related to the state of systemic immunosuppression that can be attributed both to antineoplastic treatments (chemotherapy and tumor resection surgery) and to tumor malignancy itself, as well as to the increase in cardiovascular diseases in this population. ^8 , 9^

The prevalence of cancer in epidemiological studies that describe patients with COVID-19 is rather variable. ^10 , 11^ Studies from China have shown a prevalence of 1%, which is considerably higher than the prevalence of cancer in China (0.29%). ^2^ In an analysis of 5,700 cases in New York, 6% of patients had cancer. ^11^ Grasselli et al. in an Italian cohort with 1,571 patients, demonstrated that cancer was one of the most prevalent comorbidities, accounting for 8% of all patients analyzed. ^12^

In a recently published study, when compared to patients without cancer, patients with cancer and COVID-19 were more older (63.1 years [± 12.1%] vs. 48.7 years [± 16.2]) and more often smokers (2 [22%] vs. 107 [7%]), and they had more severe alterations on chest computed tomography (CT) (17 [94%] vs. 1,113 [71%]). ^3^ No differences were observed regarding sex or other comorbidities. ^3^ Patients with cancer also had a higher rate of complications (7 [39%] vs. 124 [8%]). ^3^

Advanced age is an independent predictor of disease severity in patients with COVID-19. The median age of patients who progressed to death in Italy was 81 years (IQR 73 – 86). ^13^ Zhou et al. ^4^ observed in multivariate analysis that the risk of death were higher (OR 1.10; 95% CI 1.03 – 1.17) for each additional year. ^4^ In patients with cancer, Liang et al. observed that advanced age was the only independent predictor of the severe form of the disease (OR 1.43; 95% CI 0.97 – 2.12). ^3^ These data suggest that special attention should be given to patients with advanced age and cancer, due to the greater severity observed in this population.

In the group with history of cancer, heterogeneity was observed in association with the time interval since the last therapeutic intervention (surgery or chemotherapy). The patients who had undergone these procedures during the month prior to SARS-CoV-2 infection showed a higher incidence of serious events than cancer survivors who had not undergone therapeutic intervention, suggesting an association between the immunosuppression status caused by antitumor treatment and the increased risk of severity of COVID-19 presentation. ^3^

Initial data on mortality of patients with cancer and COVID-19 suggest that these cases have higher mortality in comparison with patients without cancer. Cohort studies from Italy have shown a prevalence of 16% to 20% of patients with active cancer among patients who died due to COVID-19. ^13 , 14^ Patients with cancer have a COVID-19 lethality rate of 7.6%, which is substantially higher than that of 1.4% in patients without comorbidities. ^9^

Knowing the risks patients with cancer face when infected with SARS-CoV-2, it is essential that they are consistently accompanied and monitored geographic areas where COVID-19 cases are on the rise. Telemedicine can aid in this monitoring. ^6^

### Choice of Oncological Treatment

Postponement of diagnostic and therapeutic interventions in patients with cancer during the pandemic period has been much discussed. Frequent visits to hospitals could increase the risk of contagion in these patients and in the healthcare team. ^6^ Social distancing measures have been encouraged in order to reduce the spread of COVID-19, given that transmission is high, and it occurs even from people who are asymptomatic. ^15^ On the other hand, it is also known that many types of cancer have higher morbidity and mortality than COVID-19, and postponement of therapy has important prognostic implications, ^16^ especially in areas where healthcare resources are typically limited and the wait for oncological care may last months. In this manner, comprehensive evaluation that takes into consideration the type of cancer, patient performance status, and the type of antineoplastic therapy required is extremely relevant to balancing the risks and benefits of possibly postponing antitumor treatment. ^17^

It is recommended to begin or continue adjuvant and neoadjuvant therapies (or any other therapy with curative potential) and to maintain therapy for metastatic diseases for which the literature has shown a clear benefit. ^6^ Oncological surgical procedures with curative potential are still recommended, always evaluating the procedure’s surgical morbidity and the possibilty of other safer neoadjuvant therapies. ^18^

Some strategies have been discussed as beneficial alternatives during the pandemic, especially for elderly patients in areas with elevated transmission. Choice of oncological therapy must consider the local options available, avoiding the need for patients to make long trips (between cities) to reach the health service. Preference should be given to oral chemotherapy, when indicated, and to endocrine therapy in sensitive tumors. ^5^

Depending on patient response, tumor type, and therapy time, it is also possible to consider spacing cycles of patients in immunotherapy from two or three to four or six weeks. ^5^ Regarding patients in radiotherapy, for treatment of more common diseases (such as breast, prostate, lung, and head cancer), reduced dose fractionation has been discussed, using a higher dose and a smaller number of sessions. This approach can lead to increased toxicity; therefore, the risks and benefits of hypofractionation should always be discussed. ^19^

It is important to discuss and weigh each strategy with patients and their family members. The doctor-patient relationship is extremely important during this moment, and the use of telemedicine can bring this interaction closer and decrease patient exposure. ^6 , 17^ It is, furthermore, recommended that, if possible, there exist health services aimed at oncological treatment that are not references for receiving patients with a COVID-19. Table 1 summarizes the American Society of Clinical Oncology’s recommendations regarding each type of specific procedure for patients with cancer. ^20^

**Table 1:** – Guidelines regarding procedures in patients in oncology during the COVID-19 pandemic

Period	Procedures	Recommendations
**Pre-diagnostic**	Oncological screening	- Routine procedures in asymptomatic patients that require visits to health centers, such as mammograms and colonoscopies, should be postponed. - Patients with suspected disease should be investigated. - Biopsies and imaging exams should be performed in patients with suspected neoplasm.
**Active oncological diagnosis**	Staging	- Comprehensive evaluation of postponement, considering diseases with indolent behavior.
	Radiotherapy	- In patients with rapidly progressing tumors or patients for whom curative radiotherapy has a clear benefit, radiotherapy should be maintained; - Some patients may have radiotherapy postponed safely; however, multidisciplinary discussion is fundamental in making individual decisions.
	Immunotherapy	- The clinical decision to postpone immunosuppressive therapies should be made in an individualized manner, taking the following factors into consideration: • Risk of recurrence of cancer in the event that there are changes to the therapy routine; • Number of therapy cycles completed; • Patient tolerance to treatment. - In patients with unstable tumors, suspending chemotherapy is not recommended.
	Chemotherapy	
	Oncological surgery	- It is necessary to routinely perform analysis of the risks of damage due to postponing surgery and the benefits of the surgery, in the midst of the COVID-19 pandemic.
	Stem cell transplant	- High-risk patients for COVID-19 whose neoplasms can be temporarily controlled with conventional therapy may have their stem cell transplant postponed. - Notwithstanding the lack of evidence regarding the transmission of SARS-CoV-2 through blood transfusion, it is prudent to test potential donors for COVID-19.
**Oncological diagnosis in remission**	Follow-up routine	- In cases of patients with low risk of recurrence who are asymptomatic during the period of follow-up, it is possible to postpone the follow-up routine in order to avoid clinical visits and possible exposure; - Telemedicine is a useful tool for evaluating patients, provided that it is carried out in a safe and regular manner; - In cases where there are recommendations regarding frequency of intervention in patients, it is prudent to postpone the intervention routine to the lowest recommended frequency.

### Prognostic Evaluation

Management of patients with cancer should include discussion of palliative care for patients with limited life expectancy. In the context of this pandemic, this discussion gains even more importance, avoiding exposure of patients with poor prognosis to contagion of COVID-19. It is important to discuss advance orders with patients who have terminal chronic diseases, always respecting local regulations, so that they do not undergo to intensive care therapy (mechanical ventilation, invasive procedures, cardiopulmonary resuscitation), in the event that this option is considered the most appropriate and proportional within the context of the underlying disease. ^21^

### Intersection Between Cancer, Cardiovascular Disease, and COVID-19

CVD and cancer are the two leading causes of death in developed and developing countries, notwithstanding significant improvements in prevention, screening, and treatment for both diseases. ^16 , 22^ With many risk factors in common, cancer and CVD often coexist in the same individuals; patients diagnosed with lung cancer, breast cancer, and colon cancer have greater risk of CVD, and patients with CVD are at a higher risk of developing many types of cancer. ^22^ The prevalence of cardiovascular risk factors in patients with cancer is elevated, with 60.4% of patients with hypertension, 23.9% with diabetes, and 22.4% with dyslipidemia. ^23^

Management of patients with cancer and CVD in oncological treatment is multidisciplinary, with the goals of controlling risk factors, reducing cardiovascular complications, and minimizing unnecessary interruptions in oncological treatment. Antineoplastic therapies are potentially toxic to the heart, and the incidence of cardiovascular complications in survivors is high. ^24^ The interaction between oncological specialists, both clinical and surgical, and the cardio-oncology service is fundamental to adequate management of these patients. ^23 , 25^

This multidisciplinary care is even more significant in patients with SARS-CoV-2 infection, which aggregates considerable morbidity and mortality. The intersection between the three diseases extends from controlling risk factors to managing pulmonary and cardiovascular complications, in addition to adjusting oncological treatment and preventing thromboembolic events. Figure 1 illustrates the main points of intersection between the three diseases. Knowledge and early implementation of appropriate therapeutic measures can result in improved prognosis for patients with cancer.

**Figure 1:** – Intersection between cancer, the heart, and COVID-19. Hypertension, advanced age, diabetes, obesity, and smorkers use are risk factors shared by these three diseases. Management of cancer in COVID-19 aims to avoid unnecessary visits to healthcare services, given that patients in oncological treatment are immunosuppressed, and they are more susceptible to developing complications of COVID-19. The most severe forms of COVID-19, manifested by hypoxemia and ARDS, result from the systemic inflammatory process, with increased inflammatory cytokines and pro-thrombotic factors. Cardiovascular complications, such as thrombosis, arrhythmias, myocarditis, and ventricular dysfunction, are frequent in this population. ACE2: angiotensin-converting enzyme 2; ARDS: acute respiratory distress syndrome; IL-6: interleukin-6; LVEF: left ventricular ejection fraction; SIRS: systemic inflammatory response syndrome. Chemo: chemotherapy; Radio: radiotherapy; Immuno: Immunotherapy. BNP: brain natriuretic peptide.

### COVID-19 and Cardiovascular Diseases

The presence of pre-existing CVD has been indicated as one of the main risk factors for the occurrence and severity of COVID-19 (9). A recently published meta-analysis, including a total of 1,527 patients, observed that, in patients with COVID-19, the prevalences of hypertension, cardiac and cerebrovascular diseases, and diabetes were 17.1%, 16.4%, and 9.7%, respectively. ^26^ In a cohort of 5,700 patients from the United States, Richardson et al. observed that the most prevalent comorbidities were hypertension (3,026 [56.6%]), obesity (1,737 [41.7%]), and diabetes (1,808 [33.85%]). ^11^ In an Italian cohort with 1,591 patients, hypertension (509 [49%]) was the most prevalent comorbidity, followed by CVD (223 [21%]), dyslipidemia (188 [18%]), and diabetes (180 [17%]). ^12^

In addition to having higher prevalence, these patients are more predisposed to developing severe forms of the disease and progressing to death. In a study from China, the mortality of patients with CVD was 10.5%; in patients with diabetes it was 7.3%, and in those with hypertension it was 6.0%. These rates are higher than those in patients without comorbidities (2.3%). ^27^ Of patients who died in Italy, 3.6% did not have comorbidities; 14.4% had a single comorbidity; 21.1% had two, and 60.9% had three or more. Hypertension (69.1%), diabetes (31.7%), and CVD (27.5%) were the most prevalent comorbidities. ^13^ In a study published by Jianfeng Xie et al., of 168 patients who died of the new coronavirus **,** 74.4% had some comorbidity. ^28^ The most frequent comorbidities were hypertension (50%), diabetes (25%), and ischemic heart disease (18.5%). ^28^

SARS-CoV-2 causes damage to the cardiovascular system through different mechanisms. The virus uses the membrane protein angiotensin-converting enzyme 2 (ACE2) to initiate viral binding with the host. ^29^ ACE2 negatively modulates the renin-angiotensin-aldosterone system by converting angiotensin 2 to angiotensin 1-7, which is opposed to the action of the angiotensin-converting enzyme. ^30^ It is highly expressed in pulmonary and heart tissues, and it performs important functions of cardiovascular and pulmonary protection. ^9 , 31^ Viral binding to this membrane protein causes inhibition of these protective mechanisms, which can result in myocardial inflammation, pulmonary edema, and acute respiratory failure. ^32 , 33^

Cardiovascular injury may also result from the systemic inflammatory response, which leads to the phenomenon of a cytokine storm. In this sense, in more severe cases, infection would result in unbalanced response by Th1 and Th2 cells. ^34^ Elevated interleukin-6 (IL-6) has been indicated as a predictor of mortality in COVID-19, suggesting that hyperinflammation in response to SARS-CoV-2 infection is an important factor for mortality. ^35^

The main cardiovascular complications resulting from COVID-19 are myocardial injury, heart failure, myocarditis, cardiac arrhythmias, shock, and coronary failure. ^36^ In some studies, elevated troponin alone was an independent predictor of mortality (OR = 26,909, 95% CI 4,086 – 177,226). ^37^ Guo et al., observed that, of 187 patients with COVID-19, 52 (27.8%) had myocardial injury, and mortality was markedly higher in patients with elevated troponin T levels than in those with normal troponin T levels (59.6 % vs. 8.9%). ^38^ Increased D-dimer levels also have a direct relation with mortality. ^4^ Myocardial injury was related to severe manifestations of the disease, with greater need for admission to the intensive care unit (ICU), ventricular dysfunction, and shock. ^4 , 39^

Cases of ventricular dysfunction observed in patients with COVID-19 result from acute coronary syndrome (ACS), myocarditis, and takotsubo syndrome. ^40 , 41^ ACS with ST segment elevation in patients with COVID-19 occurs, even without obstructive coronary artery disease (CAD), and it requires comprehensive evaluation of these patients with evaluation of ventricular function and biomarker levels. In a cohort with 18 patients, 10 (55%) had ST segment elevation without obstructive coronary disease, and, in these patients, mortality reaches 90%, possibly due to inflammation, vasospasm, hypoxia, and microcirculatory thrombosis. ^42^

Cardiac arrhythmias in COVID-19 may be a consequence of acute myocarditis, myocardial injury, and side effects of pharmacological therapy. ^38 , 39 , 43^ In a study conducted with 138 hospitalized patients, 16.7% had arrhythmias, which were more prevalent in patients admitted to the ICU (44.4% vs. 6.9%). ^39^ Guo et al., ^38^ observed that 11.7% had malign arrhythmias (ventricular tachycardia or ventricular fibrillation). ^38^ These arrhythmias were significantly more frequent in patients with elevated serum levels of cardiac troponin T and N-terminal prohormone of brain natriuretic peptide. ^38^

As COVID-19 is a recent disease, it is not clear what its long-term cardiac consequences might be. Nevertheless, a 12-year follow-up study found that survivors of SARS-CoV infection showed increased rates of cardiovascular abnormalities and lipid and glucose metabolism disorders. ^44^

Although the mechanisms of these alterations have yet to be explained, due to the structural similarity between SARS-CoV and SARS-CoV-2, the possibility is raised that COVID-19 may have similar long-term implications. ^45^

### Clinical Management of Patients with Cancer and Cardiovascular Diseases in Areas with High Transmission of COVID-19

**Prevention.** Prevention measures are a fundamental step in caring for patients with cancer and CVD during the COVID-19 pandemic. The association of CVD and cancer increases the risk of COVID-19 and the severity of the disease. Considering all aspects related to quarantine, social isolation, and distancing, cardiovascular prevention should be reinforced in these patients, implementing measures to control blood pressure, blood glucose, and lipid levels, adopting an adequate diet, and encouraging physical activity.

As there is still no vaccine that has proven effective for SARS-CoV-2, it is recommended to update influenza and antipneumococcal vaccines as a means of decreasing the incidence of concurrent infection. ^5 , 46^

Important measures to reduce the spread of the virus should be emphasized even more in this population, including correct hand hygiene with soap or alcohol gel, as well as recommendations on avoiding contact with symptomatic people and agglomerations. The Brazilian Ministry of Health, in agreement with the WHO, advises everyone to wash their hands and objects that are used frequently, to minimize sharing of objects, and to avoid touching their eyes, mouth, and nose. ^47^

In high-risk areas, measures must be taken to minimize contact and exposure in the most vulnerable cases. Accordingly, monitoring and visiting patients with CVD and/or stable cancer can be carried out with the aid of telemedicine, thus avoiding transmission of SARS-CoV-2 to this risk group. ^48^ Telemedicine has recently been regulated by the Brazilian Federal Council of Medicine during the pandemic, and it should be used in these patients in order to reduce frequent visits to healthcare services. Consultations can take place via telephone or video, aiming especially at controlling cardiovascular risk factors (such as hypertension, diabetes, smoking, obesity, and others) and monitoring symptoms.

For patients with a recent history of stem cell transplantation, self-isolation can be an effective way to avoid exposure in high-risk areas. People in contact with these patients also need to adhere to the necessary precautionary measures, minimizing exposure and obtaining tests for SARS-CoV-2 when they have flu-like symptoms.

Due to immunological changes, patients undergoing treatment for neoplasms may present an atypical manifestation of COVID-19, with milder symptoms masking severe conditions. Patients in oncological treatment and their physicians should pay close attention to any symptoms.

Prevention measures also involve organizing health services for the correct care of these patients. Ideally, hospitals for patients with cancer should not be the same as those for cases with suspected or confirmed COVID-19. If this is possible, it is recommended that there exist a service dedicated to attending these patients. ^47 , 49^ In the event of suspected COVID-19, care should be prioritized for these patients, because they show rapid progression to severe forms. ^3^ If there is no referral service for these patients, it is recommended to organize a safe flow of care for these patients in clinics and hospitals, with the adoption of all prevention measures.

It is also important for health professionals involved in caring for these patients to practice appropriate caution in relation to prevention measures. Use of personal protective equipment, hand hygiene before and after contact with patients or materials for patient use, and caution when handling medication that will be given are all recommended ( Figure 2 ). ^6^

**Figure 2:** – Prevention measures for the general population in areas with high risk of SARS-CoV-2 infection. SARS-CoV-2: severe acute respiratory syndrome coronavirus 2.

Patients with cancer are already under a great deal of psychological stress as a result of the diagnosis in itself. These patients should receive clear information and support from family members, and they are encouraged to maintain a healthy lifestyle during quarantine, with physical exercise and a healthy diet. This can help reduce anxiety and emotional stress. Social distancing measures are important to prevent the spread of COVID-19; they, may, however compromise the mental health of patients with anxiety. ^50^ A recent study on the psychological impact of quarantine indicates the psychological strain on patients who are not able to take part in social life. ^51^ It is important to emphasize that all patients with cancer who have emergencies or complications related to cancer or the cardiovascular system should seek emergency service immediately, thus avoiding poor results related to concerns regarding seeking healthcare.

### Conduct in Relation to Diagnostic Exams and Therapy in Patients with Cancer

*Diagnostic exams*: Patients with cancer frequently require exams for diagnosis, staging, and evaluation of therapeutic response. Monitoring varies depending on oncological diagnostic, but it is often necessary to perform serum tumor markers, CT, bone scintigraphy, and positron emission tomography (PET).

Nuclear medicine exams (scintigraphy and PET) take a long time, and several professionals are involved. The exposure of patients and health professionals to the contagion of COVID-19 is, therefore, relatively high, suggesting that the indication for these exams during the pandemic period should be discussed and individualized, assessing the risks and the benefits for each patient. It is recommended to postpone exams for at least 14 days patients with suspected or confirmed SARS-CoV-2 infection. ^52^

CT should be prioritized in the current context of evaluating COVID-19 infection and the presence of pulmonary thromboembolism. ^49^ Tomography findings in COVID-19 are variable; reticular images and peripheral and bilateral ground-glass infiltrates are the most common. ^49^ It is recommended to avoid transesophageal echocardiography and pulmonary scintigraphy during the pandemic, due to the high risk of contamination. ^7 , 52^ Cardiac magnetic resonance imaging should be performed after careful evaluation of the patient and the clinical indication given that both the risk of contagion and the cleaning of the equipment are more complex, due to the longer duration and the characteristics of this type of exam. ^53^ The United States Center for Disease Control recommends caution when indicating this type of investigation, and international societies recommend that its performance adhere to strict protocols. ^53^

During the pandemic period, for asymptomatic patients who are in remission, without active oncological disease, in it recommended to reschedule control exams to an appropriate moment, after the pandemic. ^6^ Moreover, priority should be given to exams for patients with urgent indications and patients who need to begin treatments with curative potential. ^6^ For patients over the age of 65 years or patients with comorbidities and with immunosuppression factors (neutropenia, hematological neoplasms, chemotherapy during the past 30 days, transplants and use of immunosuppressive medications), care should be prioritized, thus avoiding long waits, which would further overburden health services. ^53 , 54^

*Screening for cardiotoxicity:* Patients undergoing oncological therapy are at potential risks of developing cardiotoxicity. The spectrum of manifestations of cardiotoxicity is wide, with the main types being heart failure, arrhythmias, thrombosis, and CAD. ^55^ Cardiac imaging exams are essential to correct diagnosis and management of patients, especially in patients indicated for anthracycline and trastuzumab use. Diagnosis of cardiotoxicity due to these drugs is classically defined as a drop of > 10 percentage points in ejection fraction to values below 50%.

Over the past years, several strategies for monitoring and screening for early toxicity, with diagnosis still in the subclinical phase, have been incorporated into clinical practice in order to prevent these patients from developing ventricular dysfunction and heart failure. ^55 - 58^ Lopez-Sendon et al. identified a 37% incidence of cardiotoxicity in patients who previously underwent therapy with high cardiotoxic potential and who received cardiac monitoring during chemotherapy. ^59^ Nonetheless, during the current pandemic scenario, given the potential risk of COVID-19 contamination in patients with cancer in chemotherapy, it is possible to reconsider indicating these exams, reorganizing and rescheduling them in a personalized manner. ^60^

The main risk factors of cardiotoxicity are high doses of anthracycline (> 250 mg/m ^2^ ), concomitant use of cardiotoxic therapies (radiotherapy, cyclophosphamide, trastuzumab, immunotherapy, and anthracyclines), prior heart disease, the presence of two or more cardiovascular risk factors, and age < 18 or > 65 years. ^55^ The presence of signs and symptoms of heart failure is also indicative of risk of cardiotoxicity. Thus, in patients with high risk of cardiotoxicity, cardiovascular investigation should be considered.

When cardiotoxicity is suspected, evaluation of ventricular function in these patients should be conducted, preferably with transthoracic echocardiography, with exams carried out in a manner that focuses on clarifying the question. It is important to use personal protective equipment to reduce the risk of viral transmission during the exam. ^61^ Study of cardiac biomarkers may be considered in the event the patient has a visit scheduled to collect exams during chemotherapy. ^61^ Otherwise, it is not recommended to routinely solicit biomarkers to investigate cardiotoxicity. ^62^

Other imaging exams (coronary angiotomography, cardiac magnetic resonance, and coronary angiography) may be necessary for additional investigation of the etiology of ventricular dysfunction, given that cardiotoxicity is a diagnosis of exclusion, and cardiovascular complications are frequent in patients with cancer and COVID-19. ^9^ In the context of excluding CAD, tomography can be particularly useful and thus dispense with the need for further exams, where the risk of contamination and the complexity are greater. Investigation is recommended only in cases where the exam would promote a change in medical conduct. Figure 3 illustrates the recommendations that aim to assist in rational management of cardiotoxicity in these patients during the pandemic.

**Figure 3:** – Recommendations for managing cardiotoxicity during the pandemic. *Cardiomyopathy, moderate to important valvular heart disease, arrhythmia, and coronary artery disease with prior revascularization. #Age > 60 years, arterial hypertension, tobacco use, obesity, and dyslipidemia. CCTA: coronary computed tomography angiography; CMR, cardiac magnetic resonance; CVRF: cardiovascular risk factors; TTE: transthoracic echocardiography.

*Medical Therapy:* For patients with prior CVD and patients who have been diagnosed with cardiotoxicity (ventricular dysfunction), the use of angiotensin converting enzyme inhibitors and angiotensin II blockers is indicated. It has been postulated that these drugs can increase the expression of ACE2 receptors, facilitating viral entry into host cells. However, more recent evidence has not confirmed these findings, and it does not suggest that these medications increase the risk of COVID-19 infection. It is recommended that these medications should be maintained in patients with COVID-19, in the absence of contraindications. ^63 - 67^ The use of these medications is not recommended as primary prophylaxis of cardiotoxicity.

Treatment of patients with COVID-19 should take into account the clinical presentation and the presence of signs and symptoms of severity, such as hypoxemia and dyspnea. Several drugs have been tested in these settings, such as immunomodulators, antiviral, antibiotics, corticosteroids, and Il-6 and interferon inhibitors; nonetheless, there is still no definitive data on the efficacy and safety of these drugs in COVID-19. ^68^

Initial studies suggested a potential benefit from the use of chloroquine and hydroxychloroquine in COVID-19, as they have the ability to increase the endosomal pH of cells and reduce the replication of SARS-CoV-2. ^69 , 70^ Nonetheless, further studies failed to corroborate the benefit to using these drugs, and, furthermore, they have suggested an increased risk of mortality in patients who receive chloroquine. ^71 , 72^ Accordingly, the Brazilian Ministry of Health, due to the merely incipient evidence on the benefits of these drugs, has suggested that they may be used in confirmed cases and at medical discretion, as an adjuvant therapy for treating severe forms in hospitalized patients, without neglected other measures of support. ^73^

Hemophagocytic lymphohistiocytosis (HLH) is a hyperinflammatory syndrome that is still underdiagnosed in patients with a COVID-19. This syndrome is characterized by a potentially fatal state of hypercytokinemia that leads to multiple organ dysfunction. ^74^ The main findings of HLH include fever, cytopenias, and hyperferritinemia; pulmonary involvement (including acute respiratory distress syndrome) occurs in approximately 50% of patients. ^74^

In patients with COVID-19, those who progressed to death had more elevated levels of ferritin and Il-6, suggesting that inflammation is related to mortality in these patients. ^75^ In this manner, in the hyperinflammation observed in COVID-19, immunosuppression appears to be beneficial. Therapeutic options include steroids, intravenous immunoglobulin, selective cytokine blockade (for example, anakinra or tocilizumab) and Janus Kinase inhibition. ^74^

### Prospects

It is expected that, as knowledge regarding the infection by the new coronavirus evolves, we will increasingly understand the behavior of the disease in patients with comorbidities such as cancer and CVD. Prospects for effective treatment are very positive, and, regardless of the time required for science to pave the way to better results, oncologists and cardiologists must further strengthen the line of care for patients with cancer. Cardiovascular prevention, adequate treatment of cancer and CVD, and comprehensive analysis of diagnostic and therapeutic strategies are all essential in order to obtain good results in this population while facing the COVID-19 pandemic.

## Conclusion

The intersection between cancer, the heart, and COVID-19 is complex, and the rationalization of care for these patients is based on interactions between specialties and personalized conduct. Evaluation of the risks and benefits of carrying out therapeutic and diagnostic interventions requires individualized attention, considering oncological prognosis and the risk of contagion of COVID-19, especially in regions with high transmission. Patients with cancer and CVD show more severe forms of SARS-CoV-2 infection, and, for this reason, we should reinforce prevention measures even further in these patients.
